# The mosquito electrocuting trap as an exposure-free method for measuring human-biting rates by *Aedes* mosquito vectors

**DOI:** 10.1186/s13071-020-3887-8

**Published:** 2020-01-15

**Authors:** Leonardo D. Ortega-López, Emilie Pondeville, Alain Kohl, Renato León, Mauro Pazmiño Betancourth, Floriane Almire, Sergio Torres-Valencia, Segundo Saldarriaga, Nosrat Mirzai, Heather M. Ferguson

**Affiliations:** 1 Institute of Biodiversity, Animal Health and Comparative Medicine, University of Glasgow, Glasgow, G12 8QQ UK; 2 MRC-University of Glasgow Centre for Virus Research, Glasgow, G61 1QH UK; 3 Laboratorio de Entomología Médica & Medicina Tropical (LEMMT), Universidad San Francisco de Quito, Quito, 170901 Ecuador; 4 School of Engineering, University of Glasgow, Glasgow, G12 8QQ UK; 5 Bioelectronics Unit, University of Glasgow, Glasgow, G12 8QQ UK

**Keywords:** Zika, Dengue, Chikungunya, Arbovirus, Host-seeking, *Aedes aegypti*, Mosquito electrocuting trap, BG sentinel trap, Vector surveillance, Ecuador

## Abstract

**Background:**

Entomological monitoring of *Aedes* vectors has largely relied on surveillance of larvae, pupae and non-host-seeking adults, which have been poorly correlated with human disease incidence. Exposure to mosquito-borne diseases can be more directly estimated using human landing catches (HLC), although this method is not recommended for *Aedes-*borne arboviruses. We evaluated a new method previously tested with malaria vectors, the mosquito electrocuting trap (MET) as an exposure-free alternative for measuring landing rates of *Aedes* mosquitoes on people. Aims were to (i) compare the MET to the BG-sentinel (BGS) trap gold standard approach for sampling host-seeking *Aedes* vectors; and (ii) characterize the diel activity of *Aedes* vectors and their association with microclimatic conditions.

**Methods:**

The study was conducted over 12 days in Quinindé (Ecuador) in May 2017. Mosquito sampling stations were set up in the peridomestic area of four houses. On each day of sampling, each house was allocated either a MET or a BGS trap, which were rotated amongst the four houses daily in a Latin square design. Mosquito abundance and microclimatic conditions were recorded hourly at each sampling station between 7:00–19:00 h to assess variation between vector abundance, trapping methods, and environmental conditions. All *Aedes aegypti* females were tested for the presence of Zika (ZIKV), dengue (DENV) and chikungunya (CHIKV) viruses.

**Results:**

A higher number of *Ae. aegypti* females were found in MET than in BGS collections, although no statistically significant differences in mean *Ae. aegypti* abundance between trapping methods were found. Both trapping methods indicated female *Ae. aegypti* had bimodal patterns of host-seeking, being highest during early morning and late afternoon hours. Mean *Ae. aegypti* daily abundance was negatively associated with daily temperature. No infection by ZIKV, DENV or CHIKV was detected in any *Aedes* mosquitoes caught by either trapping method.

**Conclusion:**

We conclude the MET performs at least as well as the BGS standard and offers the additional advantage of direct measurement of *per capita* human-biting rates. If detection of arboviruses can be confirmed in MET-collected *Aedes* in future studies, this surveillance method could provide a valuable tool for surveillance and prediction on human arboviral exposure risk.
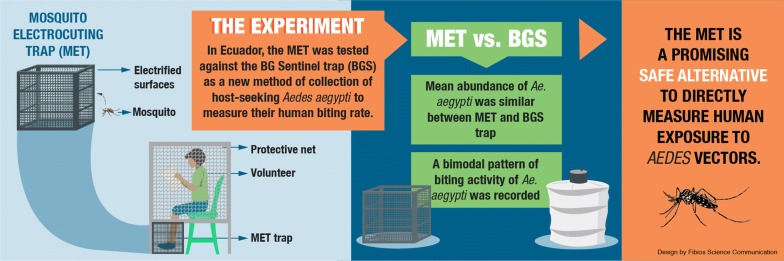

## Background

Mosquito-borne viruses (arboviruses) are an important cause of diseases in humans and animals. In 2017, estimates suggested that mosquitoes were responsible for approximately 137 million human arboviral infections with dengue (DENV), chikungunya (CHIKV) and Zika virus (ZIKV) being the most important [1]. Arbovirus transmission to humans depends on multiple factors that involve spatial movement and immunity of human populations [2–4], socio-economic factors and access to basic services (especially water) [5, 6], and the ecology and distribution of the mosquito vectors that transmit them [7–9]. These factors combine to determine the distribution and intensity of arboviral transmission and generate often complex and highly heterogeneous patterns of exposure and infection [10, 11]. As safe and effective vaccines for DENV, CHIKV and ZIKV are not yet available [12–14], control of the *Aedes* mosquito vectors remains a primary strategy for reducing transmission [15–17].

Knowledge of where and when humans are at greatest risk of exposure to infected mosquito bites is vital for prediction of transmission intensity and effective deployment of vector control [18–20]. In the case of malaria, this information is used to estimate a time or site-specific “Entomological Inoculation Rate” (EIR); defined as the number of infected mosquito bites a person is expected to receive. This metric is usually derived from conducting human landing catches (HLCs); a method in which a participant collects and counts the number of mosquito vectors landing on them over a given sampling period, then the sample is tested for the presence of a pathogen [21]. By providing a direct estimate of human exposure, the HLC provides sensitive predictions of malaria transmission [19, 22–24]. However, this method raises ethical concerns due to the requirement for human participants to expose themselves to potentially infectious mosquito bites [25]. In the case of malaria, this risk can be minimized by providing participants with prophylaxis [26]. However, such remediation is not possible for arboviruses where often no prophylaxis is available, and therefore HLCs are not recommended for the surveillance *of Aedes*-borne arboviruses [27, 28].

Standard entomological monitoring for *Aedes* vectors is usually based on “exposure-free” surveillance of larvae or non-biting adults. This includes surveys of larvae or pupae in water containers [29, 30], and collection of adult mosquitoes resting inside and/or around houses to indirectly estimate human-vector contact rates [29, 31]. While such surveillance methods are useful for confirming vector abundance and distribution, they are poor predictors of epidemiological outcomes such as disease incidence and outbreak potential [32, 33]. Consequently, there is a need for vector sampling methods that can provide more reliable entomological indicators of arboviral transmission.

Human exposure to arboviral infection is likely best assessed by surveillance of “host-seeking” (human-biting) *Aedes* mosquitoes. Several methods have used to sample host-seeking *Aedes* including a variety of fan-operated traps that use visual attraction cues (e.g. Fay [34], the Fay-Prince trap [35], the black cylinder suction trap [36], duplex cone trap [37]) and lure-based traps. For the latter, artificial odours and attractants have been developed and tested for use in traps such as kairomone blends [38, 39], BG-Lure® cartridges [40, 41] and carbon dioxide (CO_2_) [42]. Additionally, other trapping methods have been developed that use live hosts as lures (e.g. animal-baited traps [43] and human-baited traps [44, 45]). Only a few studies have directly compared such alternative trapping methods against the HLC with most being outperformed by the latter [44, 45]. Out of all these methods, the BG-sentinel (BGS) trap has been demonstrated as one of the most effective and logistically feasible [46, 47], and thus often considered a gold standard for *Aedes* surveillance [48, 49]. In a range of trap evaluation studies, the BGS outperformed other methods for *Aedes* vectors except for HLC [50]. Despite these advantages of the BGS, its ability to accurately reflect the biting rates experienced by one person remains unclear. Consequently, there is still a need for a safe alternative for direct assessment of human biting rates.

Recently, a new mosquito electrocuting trap (MET) was developed as an exposure-free alternative to the HLC for sampling malaria vectors [51–53]. This trap was built on previous work using electrified nets and grids to trap tsetse flies [54, 55] and mosquitoes [56, 57] attracted to hosts or their odours. Similar to the HLC, this sampling method also uses human participants to lure mosquito vectors and trap them. However, the MET provides participants with full protection from mosquito bites so that no exposure is required. The MET consists of four squared-shaped electrocuting surfaces that are assembled around the legs of a host, with the rest of their body being protected by netting. Host-seeking mosquitoes are attracted towards the host by odour and heat cues as normal but are intercepted and killed before landing. In previous trials in Tanzania, the MET matched the performance of the HLC for sampling malaria vectors in rural and urban settings [51–53]. This trap has also been used to assess host preference by baiting with human and livestock hosts [53], although it has not yet been evaluated for sampling *Aedes* vectors. If successful in this context, the MET could significantly improve ability to monitor and predict arboviral transmission by facilitating an exposure-free direct estimation of EIR.

This study reports the first evaluation of METs for sampling host-seeking *Aedes* vectors in a hotspot of DENV and ZIKV transmission in coastal region of Ecuador. This region is endemic for such arboviral diseases and has accounted for most of the cases reported in Ecuador. For instance, during the CHIKV outbreak in 2015, a total of 33,625 cases were reported in Ecuador, from which 96.02% was reported in the coastal region [58]. A similar pattern occurred during the ZIKV outbreak in 2016 and 2017, where approximately 98.49% of the cases were reported in this region from a total of 5303 cases [59, 60]. DENV has been reported every year in high numbers and considering 2016 and 2017, 84.78% of cases came from the coastal region from a total of 25,537 cases [60, 61].

The objectives of this study were to: (i) evaluate the performance of the MET relative to the BGS trap for sampling host-seeking *Ae. aegypti* and other mosquitoes in the study area; and (ii) use the MET to characterize the biting time of *Ae. aegypti* and other relevant mosquito species and their association with microclimatic conditions.

In addition, we took the opportunity to test for the presence of arboviruses in the collected *Aedes* females by both trapping methods to investigate arboviral transmission in the local area.

## Methods

### Location and time of the study

**Fig. 1:**
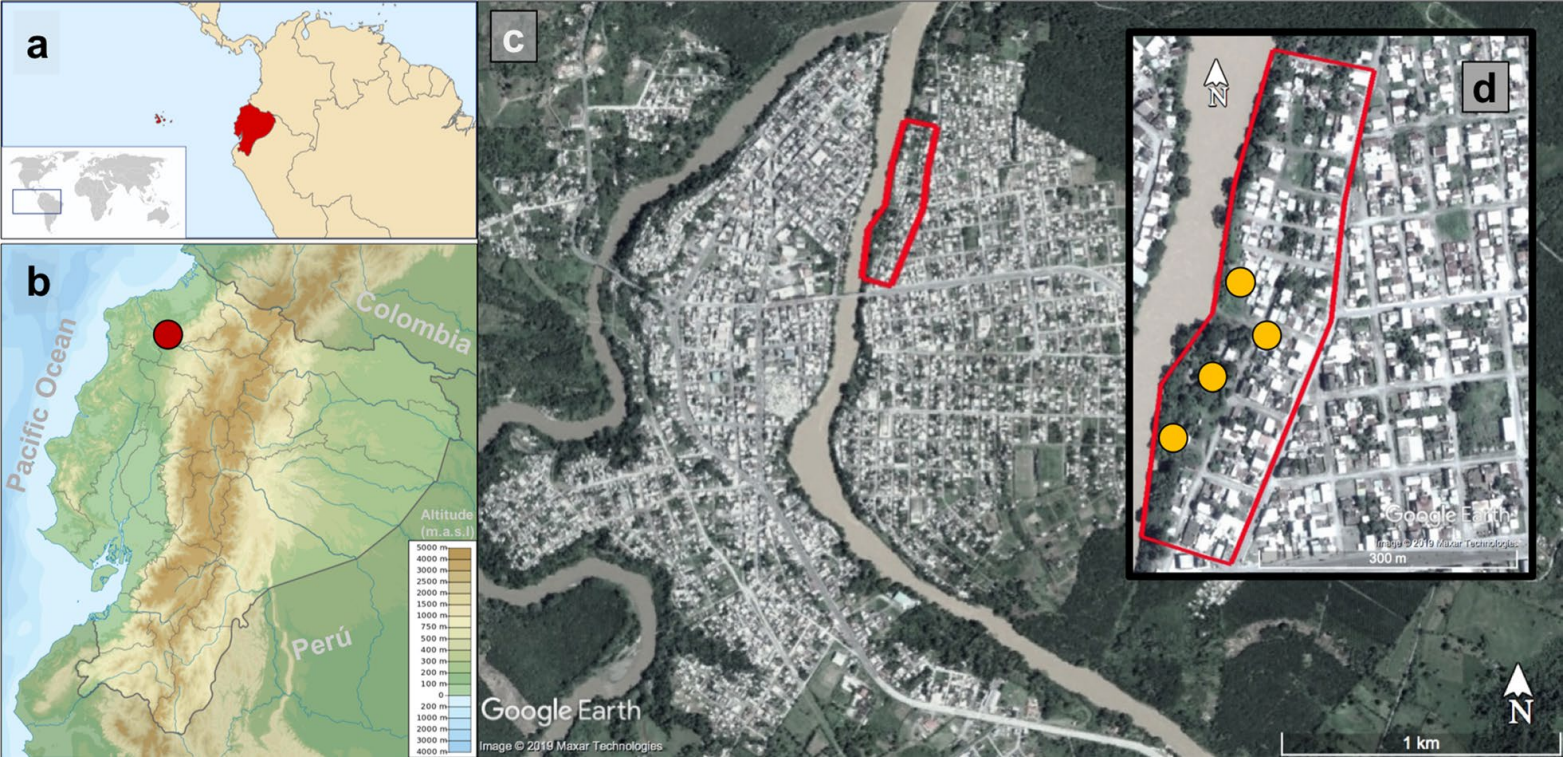
View of the urban area of the city of Quinindé. **a** Location of Ecuador in the Americas highlighted in red (taken from [96]). **b** Location of the city of Quinindé in the Pacific Coastal region, spotted by the red circle. **c** City of Quinindé showing Los Higuerones neighbourhood enclosed by the red line. **d** Enlarged view of Los Higuerones with the houses sampled spotted by the orange circles

This study was conducted in the neighbourhood of “Los Higuerones” (0°19′34″N, 79°28′02″W, 78 meters above sea level), located in the city of Quinindé (Rosa Zárate) (Ecuador). This neighbourhood is located in an urban setting dominated by small, closely packed houses (Fig. 1c), bordering the eastern side with the Blanco River (Fig. 1d). Quinindé is located in the Province of Esmeraldas, the northernmost province in the coastal region of Ecuador. During the 2015 outbreak of CHIKV, this province accounted with the highest disease burden in the country, with a total of 10,477 cases [58]. While for DENV, during 2016, Quinindé alone accounted for 52% of the cases within Esmeraldas Province, with a total of 689 cases out of a total of 1319. In 2017, the number of DENV cases in Quinindé was much lower compared with 2016, where only 87 cases were reported out of 334 in the Province of Esmeraldas. Although there is a permanent incidence of arbovirus cases along the year, a higher incidence is usually reported during the first half of the year [6].

The study was carried out across 12 days in May 2017 (4th–12th, and 16th–18th). On each day of the study, mosquito sampling was conducted over 12 h, from 7:00–19:00 h. Mosquito sampling was conducted within the peridomestic area (garden/yard) of four households (Fig. 1d). These houses were selected on the basis of being physically accessible, and having residents present and willing to participate during an initial tour of the area with a local guide. Houses were separated by approximately 90 m from one another.

### Trapping methods

Over the study period, host-seeking mosquitoes were sampled by two different methods as described below.

#### BG-Sentinel trap (BGS)

**Fig. 2:**
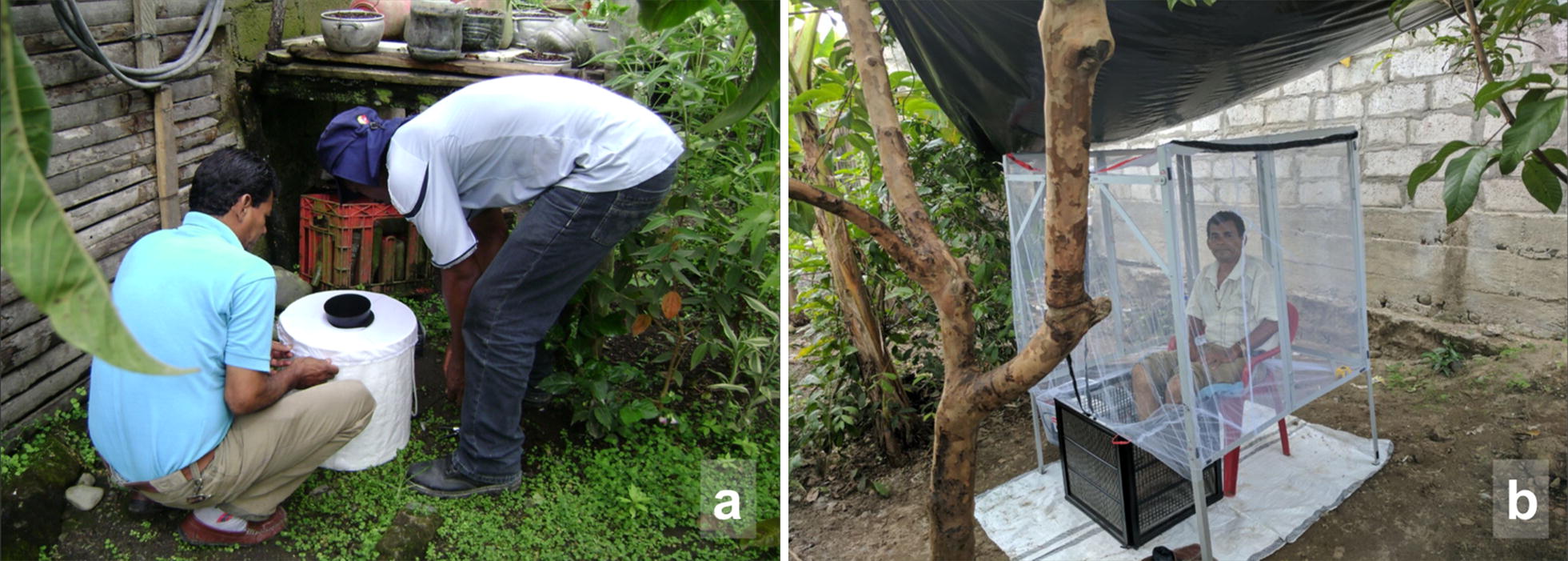
Trapping methods used in this study. **a** Typical set-up of a BGS trap. **b** Set-up of a MET with a technician luring mosquitoes

The BG-Sentinel® trap (BioGents, Regensburg, Germany) is a white, cylinder-shaped trap made of plastic with a gauze cloth covering the top and a hollow black cylinder in the top centre of the trap (Fig. 2a). The trap operates with a 12 V battery that powers an internal fan that produces inwards artificial air currents. In this study, each trap was baited with two BG-Lure® cartridges and a 1.4 l cooler bottle filled with dry ice in order to maximize the attractiveness of traps to *Aedes*; as it is known that CO_2_ increases the catch efficiency of BGS traps [46, 47, 62]. Mosquitoes are attracted towards the baited traps and then sucked through the hollow black cylinder into an internal mesh bag that can be easily removed for subsequent processing.

#### Mosquito electrocuting trap (MET)

The METs used here consisted of four 30 × 30 cm panels which are assembled into a box around the lower legs of a seated person (Fig. 2b). Each panel is made up of stainless-steel electrified wires set within a PVC frame. The wires are positioned 5 mm apart, which is close enough so that mosquitoes could not pass through without making contact. Wires are vertically arranged in parallel, alternating positive with negative. When mosquitoes try to go through, contact is made and the voltage between wires kills them.

Mosquitoes attracted towards the volunteer were intercepted and killed on contact with these panels. The MET is powered by two 12 V batteries connected in series to a power source giving a power output of approximately 6 W (10 mA, 600 V). As an additional safety feature, a protective inner panel made from wide non-conductive plastic grid was fit into each frame preventing accidental contact between users and the electrified wires.

As an additional accessory to the MET, a retractable aluminium frame was built to cover the rest of the volunteer’s body with untreated mosquito-proof netting. Thus, volunteers were completely protected from mosquito bites during their participation in trapping. A plastic tarpaulin was erected over the MET station at a height of 2 m to protect users from direct rain and sunlight. Each MET was also set up on top of a white plastic sheet to isolate it from the ground and make it easier to see and collect shocked mosquitoes that fell onto the ground after touching the MET.

### Experimental design

Every day of the study, four traps (two METs and two BGS traps) were set up in the peridomestic area of the four households (one trap per household) at the ground level under shade conditions. Traps were rotated among households each day, so that a different trapping method was used every consecutive day in each house. At the end of the study, this resulted in 6 days of trapping being conducted with each of the 2 methods at all houses.

MET collections were carried out by members of the research team, who were all adult men (30–50 years-old). During each hour of the collection period, one member sat within the MET for 45 min, with the trap being turned off for the remaining 15 min to allow volunteers to take a break. Members of the study team took turns sitting in the trap so that different collectors lured every hour. During the 15 min period when traps were turned off, mosquitoes were recovered from trap surfaces and the ground below using a pair of forceps, counted and placed in empty 15 ml falcon tubes; which were labelled with a unique code linked to the date, household ID, trap ID, hour period and collector ID. Tubes were stored in a cooler box of 45 l capacity filled with dry ice to kill, preserve and transport the specimens.

Each BGS was baited with two BG-Lure® cartridges on each day of sampling; with lures exchanged between the two BGS traps each day to minimize bias due to differential lure efficiency. BGS traps were further baited with carbon dioxide by adding one 1.2 l Coleman® polyethylene cooler bottle filled with dry ice. Dry ice containers were topped up every day. Like the MET, BGS sampling was conducted for 45 min of each sampling hour, with mosquito collection bags being checked and emptied during 15 min break periods. Mosquitoes from BGS collection bags were emptied into pre-labelled plastic bags and transferred into a cooler box with dry ice to kill and preserve the mosquitoes.

Temperature and relative humidity data were collected every 10 min at each mosquito sampling point using TinyTag® Plus 2 TGP-4500 (Gemini Co., Chichester, UK) data loggers. Data loggers at the BGS sampling stations were tied and hung inside each of the traps, and loggers at MET sampling points were placed on top of the bottom border of the netting frame, next to the MET.

### Morphological analysis

Mosquitoes collected in the field were transported to the Medical Entomology and Tropical Medicine Laboratory of the San Francisco de Quito University (LEMMT-USFQ) in cooler boxes filled with dry ice. At LEMMT-USFQ, mosquitoes were morphologically identified using taxonomic keys [63–65], counted and sorted into different cryo-vials according to date, household, trap type, hour of collection, species, sex and physiological status of females (blood-fed/gravid and non-blood-fed). All female *Ae. aegypti* specimens were retained for subsequent molecular analysis to test for the presence of ZIKV, DENV and CHIKV. These *Ae. aegypti* samples were grouped into pools of a maximum of 5 individuals.

### Molecular detection of arboviruses

All pools of female *Ae. aegypti* specimens were screened for the presence of CHIKV, DENV and ZIKV. Details on the RNA extraction, reverse-transcription and PCR procedures are given in Additional file 1: Text S1, Table S1 and Table S2.

### Data analysis

Statistical analyses were performed in R 3.5.0 and R Studio 1.1.419. Generalized linear mixed models (GLMM) were used to investigate variation in the abundance of host-seeking mosquitoes (per day and per hour) using the package *lme4* in R [66]. As mosquito abundance data were overdispersed, all models were fitted with a negative binomial distribution. For all response variables of interest as described below, model selection was carried out through a process of backward stepwise elimination from a maximal model using likelihood ratio tests (LRT) [67].

Statistical analysis was performed for *Ae. aegypti* and *Culex quinquefasciatus* as the latter was the only other mosquito species found in high abundance in the study area. *Culex quinquefasciatus* is a nuisance biting mosquito and also a known vector of West Nile virus (WNV) [68].

The BGS traps functioned continuously across all days and sampling hours. However, the METs stopped running during some sampling hours; generally, under conditions of very high humidity due to rainfall which resulted in dampness on the traps and some temporary short circuiting (e.g. observed as plumes of smoke at the bottom junction with the frames). When these malfunctions occurred, the damaged traps were turned off and repaired. This resulted in variation in the total number of hours sampled with each trapping method (MET: 229 h; BGS: 270 h). This variation in sampling effort was accounted for in the statistical analysis. Days having less than 9 h were excluded from the analysis.

Four models were built to assess the variation in the abundance of each mosquito species and sex combination, respectively. For each of these four response variables, a maximal model was constructed that included the fixed explanatory variables of sampling effort (total number of hours of collection), trap type (MET or BGS), daily mean relative humidity (%RH), and daily mean temperature (°C). In addition, the interaction between daily mean temperature with relative humidity was also included. Sampling day (1 through 12), household ID, trap ID and attractant ID (BG-Lure cartridge ID or MET volunteers ID) were included as random effects.

Mosquito biting activity was assessed through analysis of variation in the mean number of females (*Ae. aegypti* and *Cx. quinquefasciatus*) caught per hour. Here, each mosquito species was analysed separately. Each model included the explanatory variables trap type (MET or BGS), sampling hour, mean temperature (°C) per hour, mean relative humidity (%RH) per hour, and the interaction between hourly temperature and relative humidity. Sampling hour was defined as a continuous variable recoding the first hour of trapping (7:00–8:00 h) into 1, and increasing “hour” by one digit for each subsequent hour until 12 h (17:00–18:00 h). Sampling hour was fit both as a linear and quadratic term, with the latter being used to test for peaks in biting time as have been previously reported for these mosquito species [69]. In addition, sampling day, trap ID, cluster ID, household ID (nested within cluster ID) and attractant ID (BG-Lure cartridge ID or MET volunteer ID) were fitted as random effects.

## Results

### Mosquito species and abundance

During the 12 day-experiment, a total of five mosquito species were collected by both trapping methods (Table 1). *Culex quinquefasciatus* was the most abundant species (78.6%) followed by *Ae. aegypti* (15.63%), and small numbers of *Aedes angustivittatus* (2.69%), *Limatus durhami* (2.33%) and *Psorophora ferox* (0.15%). A small proportion of mosquitoes could not be identified (0.51%, Table 1). Overall, more mosquitoes were collected with the BGS trap (60.77%) than with the MET (39.23%), but the numbers of *Ae. aegypti* were relatively similar (Table 1).

**Table 1:** Abundance of mosquito species collected by MET and BGS traps

Species	Mosquito electrocuting trap (MET)				BG-Sentinel (BGS) trap				Grand total
	**♂**	♀ Unfed	♀ Fed	Total	♂	♀ Unfed	♀ Fed	Total	
*Aedes aegypti*	100	99	19	218	93	91	27	211	429
*Culex quinquefasciatus*	496	238	44	778	960	345	77	1382	2160
*Aedes angustivittatus*	4	38	6	48	0	24	2	26	74
*Limatus durhami*	0	22	0	22	0	42	0	42	64
*Psorophora ferox*	0	1	2	3	0	1	0	1	4
Unknown	0	5	3	8	0	5	1	6	14
Total				1077				1668	2745

Notes: Mosquito species abundances are split by sex and feeding status of females. The total sampling effort with the two METs was 229 h, while for BGS traps was 270 h over the 12 days of sampling

In the BGS traps, some non-target insects including house flies, butterflies, crane flies, and many fruit flies were caught. No insect taxa other than mosquitoes shown in Table 1 were caught in MET collections.

**Fig. 3:**
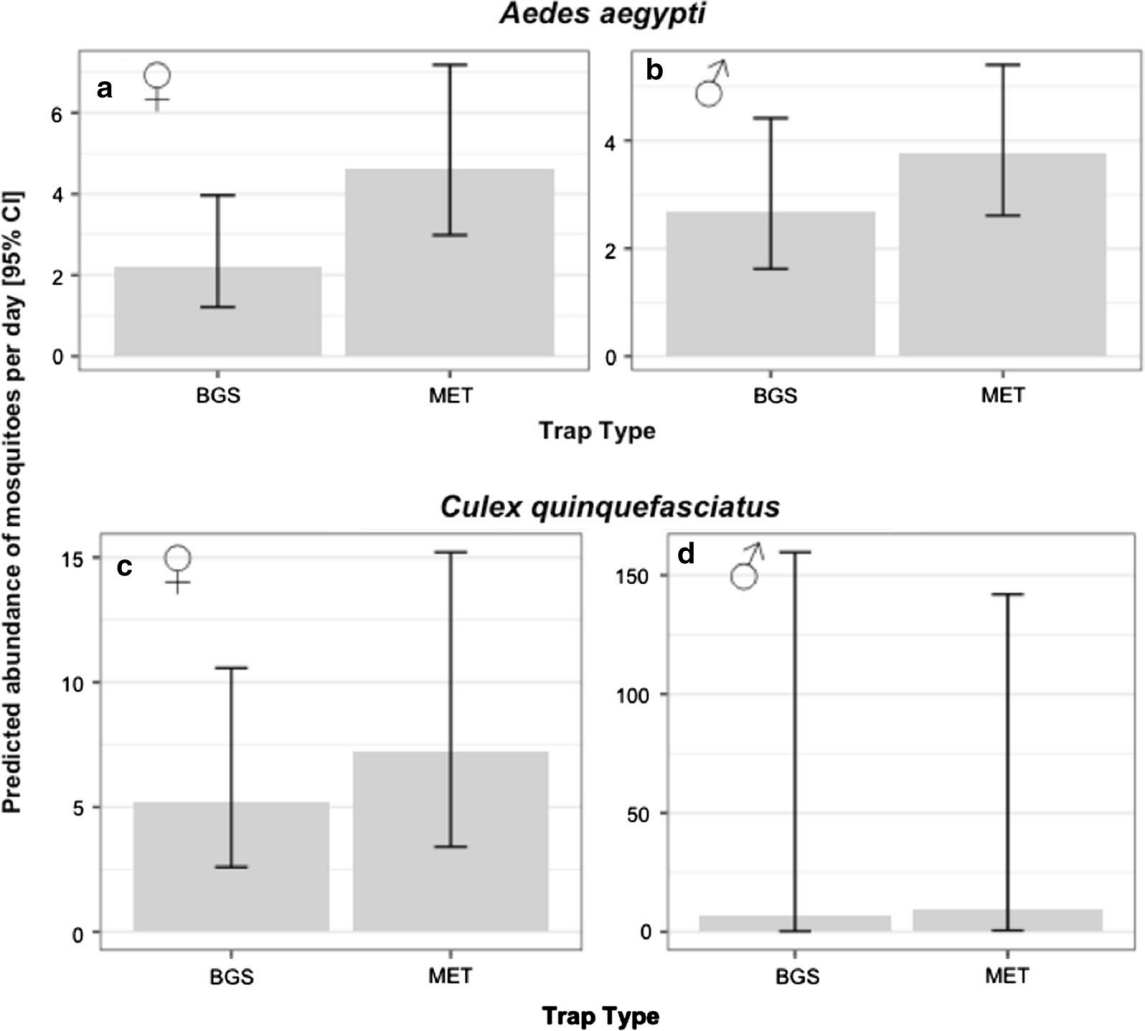
Predicted mean daily abundance of mosquitoes caught with different trapping methods. **a**, **b** Data for *Ae. aegypti*. **c**, **d** Data for *Cx. quinquefasciatus*. **a**, **c** Data for females (**♀**). **b**, **d** Data for males (**♂**). Error bars indicate the 95% confidence intervals (CI)

**Fig. 4:**
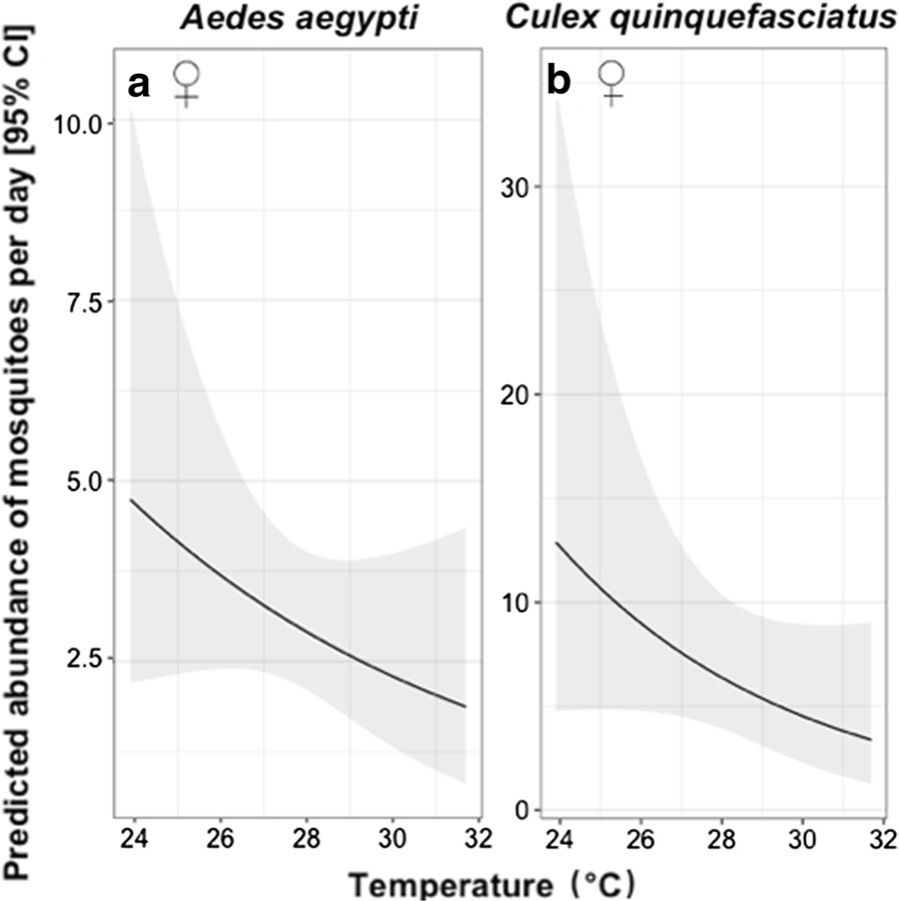
Predicted relationship between mean temperature and number of female mosquitoes collected. **a** *Ae. aegypti* females. **b** *Cx. quinquefasciatus* females. The solid line indicates the mean predicted abundance and the shaded area indicates the 95% confidence intervals (CI)

The mean daily abundance of *Ae. aegypti* was approximately 2 females and 3 males for the BGS trap, and 4 females and 4 males for the MET, but no significant differences between trapping methods were found (Table 2, Fig. 3a, b). The only significant predictor of daily abundance of females *Ae. aegypti* was temperature, which exhibited a negative association (Table 2, Fig. 4a). Similarly, the mean daily abundance of *Cx. quinquefasciatus* females did not significantly differ between trapping methods (Table 2, Fig. 3c, d); however, confidence intervals (especially for males) around estimates were very large, indicating that larger sample sizes may be required to robustly test if there were differences between trap types. The number of female *Cx. quinquefasciatus* per day varied between 16–207, with variation being even more pronounced for males where a high of 576 was caught on one day. The daily abundance of female *Cx. quinquefasciatus* was negatively associated with daily temperature (Table 2, Fig. 4b) and positively associated with the number of hours sampled in a day, while no significant differences were found in *Cx. quinquefasciatus* regarding any covariate (Table 2).

**Table 2:** Summary for the terms tested from mosquito daily abundance

Explanatory variable	*Aedes aegypti*						*Culex quinquefasciatus*					
	Males ♂			Females ♀			Males ♂			Females ♀		
	*χ*^2^	*df*	*P*	*χ*^2^	*df*	*P*	*χ*^2^	*df*	*P*	*χ*^2^	*df*	*P*
Sampling effort	3.38	1	0.07	1.95	1	0.16	0.31	1	0.58	15.91	1	< 0.001*
Trap type	2.18	1	0.14	0.60	1	0.44	0.95	1	0.33	1.5	1	0.22
Temperature	0.22	1	0.64	4.62	1	0.03*	0.06	1	0.8	6.86	1	< 0.01*
Relative humidity	1.14	1	0.29	2.17	1	0.14	1.23	1	0.27	1.1	1	0.29
Temperature × Humidity^a^	2.22	1	0.14	1.24	1	0.26	1.07	1	0.3	1.27	1	0.26

*Significant values

^a^Fixed effect indicating interaction term

Notes: Chi-square (*χ*^2^), degrees of freedom (*df*) and *P*-values (*P*) are provided for each sex within species

### Mosquito biting activity

**Fig. 5:**
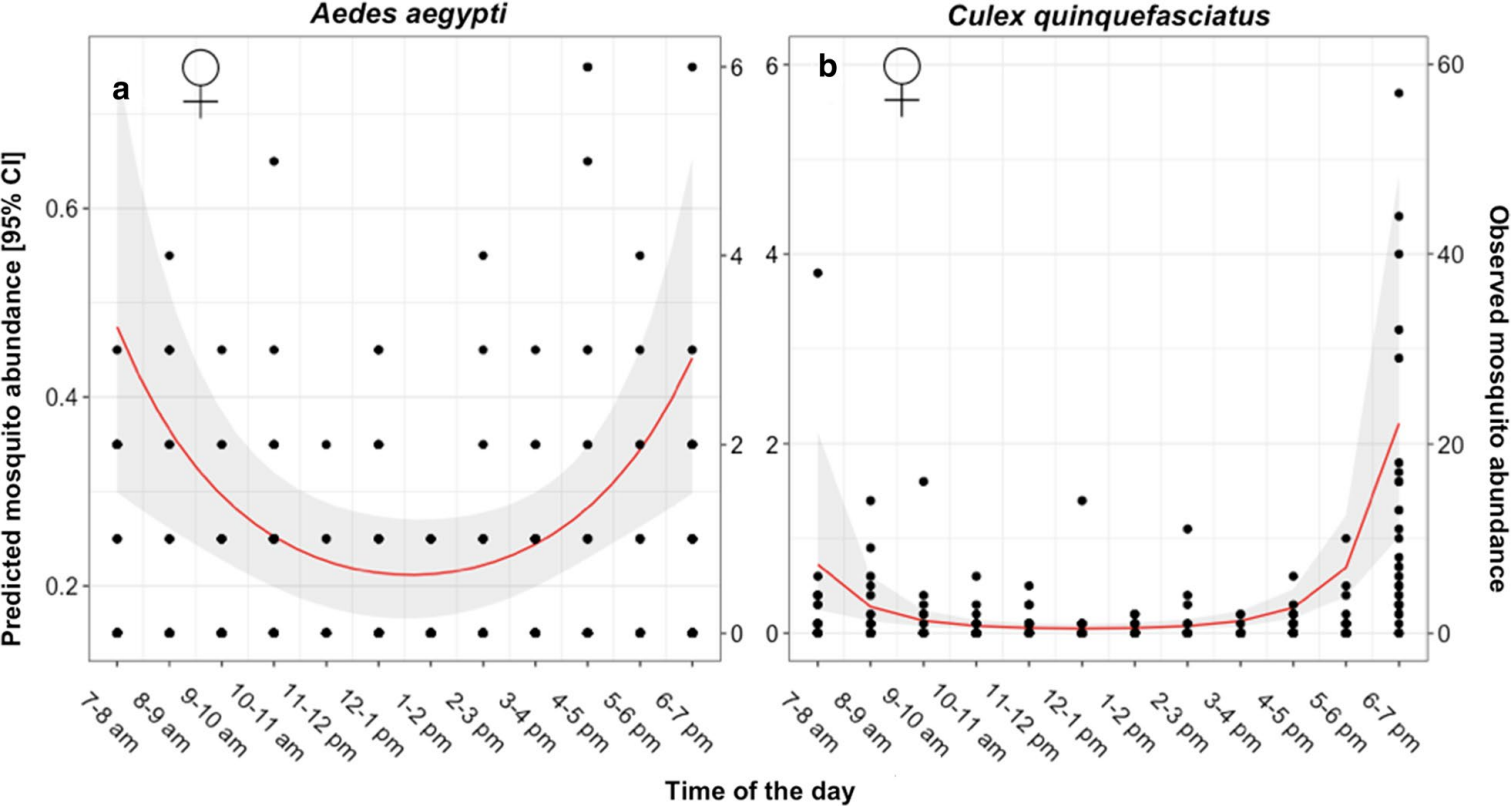
Predicted abundance of biting mosquitoes between 7:00–19:00 h. **a** *Ae. aegypti* females. **b** *Cx. quinquefasciatus* females. Dots represent the observed values which correspond to the right Y-axes. The red line corresponds to the predicted mosquito abundance and the shaded area to the 95% confidence intervals (CI); both correspond to the left Y-axes

**Fig. 6:**
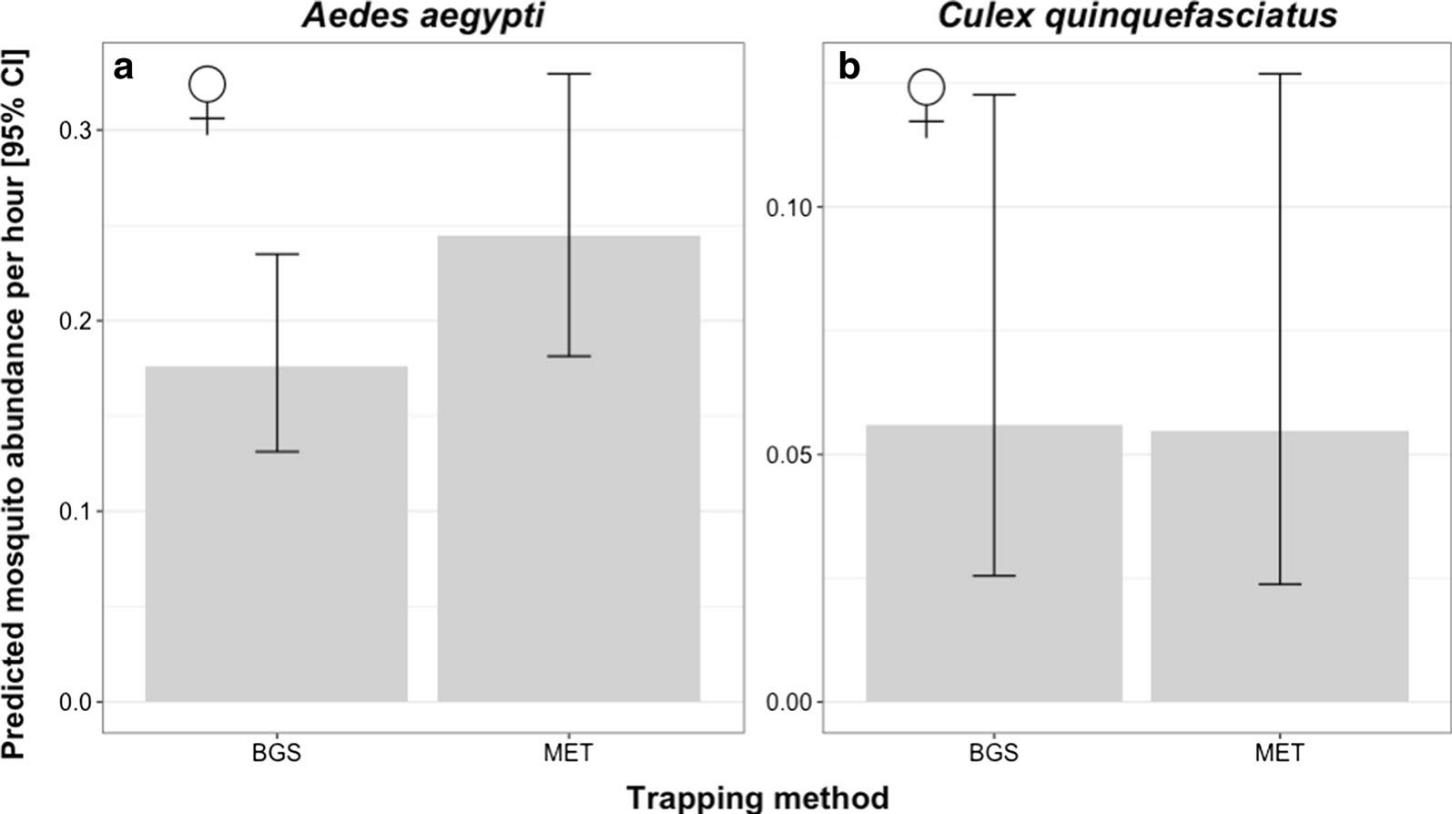
Predicted hourly abundance of mosquitoes using different trapping methods. **a** *Ae. aegypti.* **b** *Cx. quinquefasciatus*. The error bars indicate the 95% confidence intervals (CI)

**Fig. 7:**
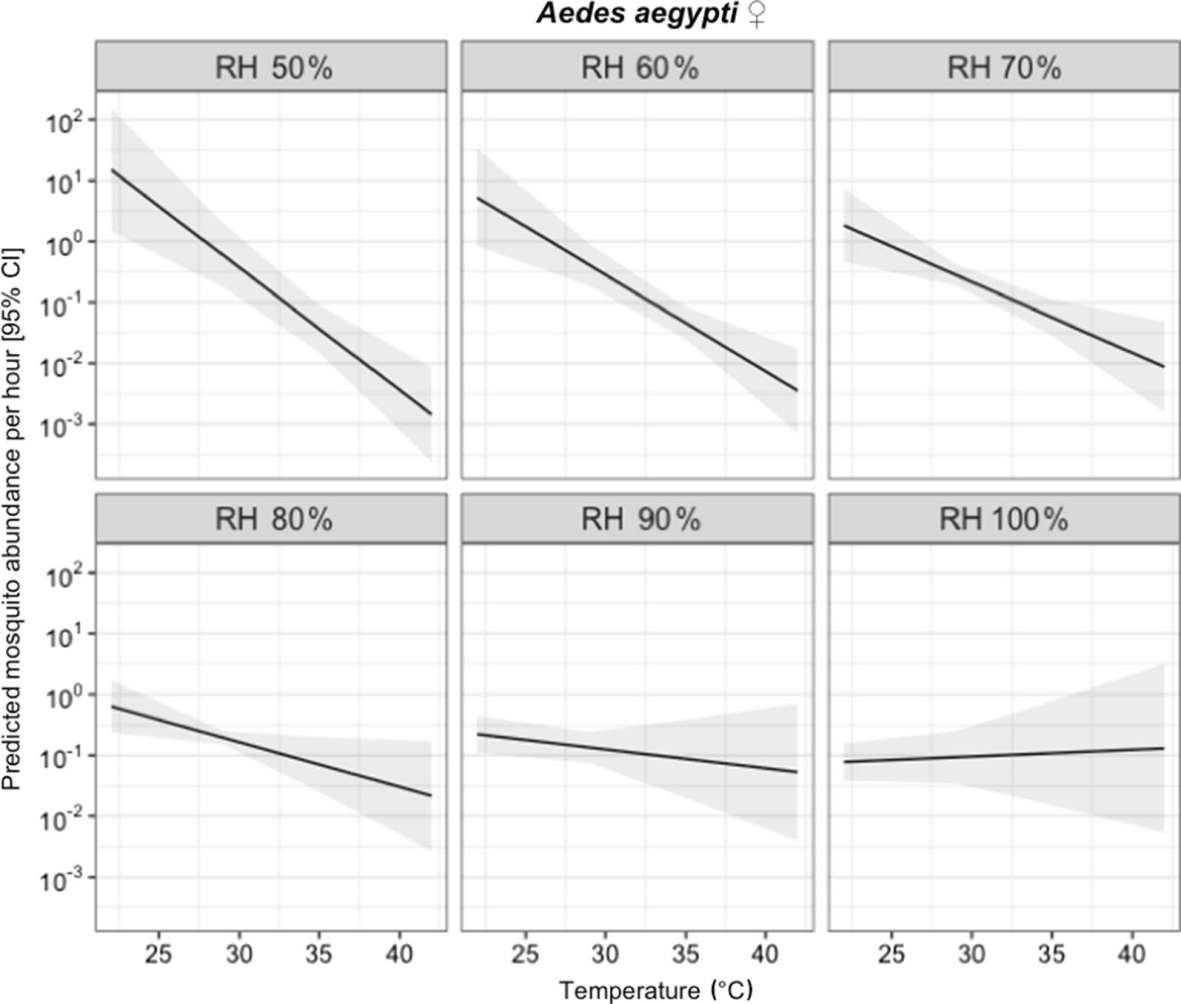
Predicted relationship between the hourly abundance of *Ae. aegypti* females and mean temperature (°C) under different relative humidity (RH) conditions. The black line represents the predicted abundance of *Ae. aegypti* in that hour, with the shaded area representing the 95% confidence intervals (CI)

Hourly mosquito catches recorded for BGS and METs were used to characterize the biting activity of female *Ae. aegypti* and *Cx. quinquefasciatus.* Variation in the hourly biting activity of female *Ae. aegypti* was best explained by a quadratic association between hourly mosquito abundance and time (Table 3), with activity being highest in the early morning and late afternoon, and little activity during the middle of the day (Fig. 5a). After taking this hourly variation in biting rates into account, there was no additional impact of trapping method on the number of female *Ae. aegypti* collected per hour (Table 3, Fig. 6). Variation in the hourly biting activity of *Ae. aegypti* was also significantly associated with an interaction between temperature and relative humidity (Table 3). This interaction arose because the number of *Ae. aegypti* caught per hour was negatively associated with temperature under conditions of low relative humidity; but the strength of this association was lower as humidity increased (Table 3, Fig. 7), although temperature and humidity were strongly associated (Additional file 2: Figure S1).

**Table 3:** Summary for the terms tested for association with female mosquito hourly abundance

Explanatory variable	*Aedes aegypti* females ♀			*Culex quinquefasciatus* females ♀		
	*χ*^2^	*df*	*P*	*χ*^2^	*df*	*P*
Trap type	0.60	1	0.44	7e-04	1	0.98
Time (linear)	na	na	na	na	na	na
Time (quadratic)	8.70	1	< 0.01*	142.1	1	< 0.001*
Temperature	na	na	na	2.07	1	0.15
Relative humidity	na	na	na	0.09	1	0.77
Temperature × Humidity^a^	6.60	1	0.01*	0.09	1	0.76

*Significant values

^a^Fixed effect indicating interaction term

*Notes*: Chi-square (*χ*^2^), degrees of freedom (*df*) and *P*-values are provided for females of each species. “na” indicates “not applicable” values for which single term significance was not possible because of their involvement in significant higher order terms

The biting activity of female *Cx. quinquefasciatus* also varied significantly across the sampling day. As with *Ae. aegypti*, this pattern was characterized as a quadratic relationship in which mosquito activity peaked during the early morning and late afternoon (Table 3, Fig. 5b). Accounting for this activity pattern, there was no difference in the number of *Cx. quinquefasciatus* caught per hour in different trapping methods (Table 3, Fig. 6b), and no association with temperature or humidity.

### Molecular screening for ZIKV, DENV and CHIKV

*Aedes aegypti* females were tested for ZIKV, DENV 1-4 and CHIKV and none of the samples were found positive. For a detailed description on the molecular results, please see Additional file 1: Text S2 and Additional files 3, 4, 5, 6, 7, 8, 9, 10: Figures S2–S9. In Additional files 4, 5, 6, 7, 8, 9, 10: Figures S3–S9, asterisk indicates the samples that had a weak band at the corresponding expected size, and ^ indicates the samples that showed a size close to the expected one. The red dashed line is positioned at the corresponding expected size for each PCR run.

## Discussion

Identifying an accurate method to predict the exposure of humans to infected mosquito vectors has been an enormous challenge for *Aedes*-borne pathogens [70, 71]. Here, we present the MET as a potential alternative for safe measurement of *Aedes* landing rates on humans. When tested in Ecuador, the MET provided similar estimates of *Ae. aegypti* abundance and biting activity as the current gold standard, the BGS sentinel method. While the BGS uses artificial odour baits and carbon dioxide (CO_2_) to lure mosquitoes into a standardized trap, the MET directly estimates the number of *Aedes* host-seeking within the immediate vicinity of a real host. The MET can also be used to measure biting rates on a range of different host species (e.g. [53]), which currently cannot be performed with the BGS and other methods. The standardization provided by the BGS makes it easy and effective to use in widescale surveillance [48, 50], although a limitation is that non-biogenic CO_2_ sources are not always available [72]. However, the degree to which BGS collections accurately reflect *per capita* human biting rates is unclear. For example, BGS trapping efficiency may vary with the type and number of lures used, rate of CO_2_ released (quantity per time), location and colour of the trap (e.g. BGS 1 and BGS 2) [38, 46, 73], making it difficult to infer how different variants translate into exposure experienced by one person in that environment. An advantage of the MET is that it is more directly analogous to the human landing catch in sampling mosquitoes in the process of host-seeking on a person and also estimate variability in attraction between individuals. This could also be seen in the total catches of the other mosquito species when compared to the total numbers trapped by the BGS. The MET could thus provide a useful supplementary surveillance method for estimation and validation of human-biting rates and the associated entomological inoculation rate (EIR).

By facilitating a safe and more direct estimation of the EIR for *Aedes*-borne viruses, the MET could provide robust and precise entomological indicators of transmission intensity [51–53]. Such indicators are much needed to understand heterogeneity in transmission [33, 74, 75] and evaluate the efficiency of vector control interventions. However, this relies on the assumption that the MET accurately reflects the true *Aedes* exposure of one person per unit of time. Estimates of human exposure to the malaria vector *An. gambiae* (*s.l.*) from the MET were similar to those of the human landing catch in some studies [53, 76], whereas in others mosquito abundance was underestimated by the MET compared to the HLC [52]. Here, it was not possible to directly compare the MET to the HLC because of ethical restrictions in using the latter in an area of high arboviral transmission. However, we speculate that one factor that could cause the MET to underestimate *Aedes* vectors biting rates is the area of the body protected. Whereas African *Anopheles* vectors generally prefer feeding on the lower legs and feet [77–79]; it is not clear if *Aedes* prefer to bite on specific parts of the body [80, 81]. As a next step in validating this approach, we recommend the MET to be directly compared to the HLC under controlled conditions with uninfected *Aedes* vectors (e.g. semi-field experiments), ideally using a defined *Ae. aegypti* strain and appropriate experimental design to act as a reference standard for future comparison.

Both the MET and BGS trap sampled a similar composition of mosquito species in the study period. However, estimates of the mean daily and hourly abundance of *Ae. aegypti* and *Cx. quinquefasciatus* were slightly but not statistically higher in MET than in BGS collections. The relatively short period of this (12 sampling days) may have limited power to detect for minor to moderate differences between trapping methods. We thus conclude the MET is at least as good as the BGS gold standard for sampling host-seeking *Aedes* vectors in this setting, but also recommend further longer-term comparisons over a wider range of seasons, sites and participants to evaluate whether the MET outperforms the BGS. If we assume that MET is equivalent to HLC, these results are also consistent to those shown by Kröckel et al. [50], who also observed that HLC captured more mosquitoes, although not statistically different from the BGS.

Mosquito collections conducted here were also used to test for associations between *Aedes* host-seeking activity and microclimatic conditions. The impact of temperature and humidity on the life history, physiology, behaviour and ecology of *Ae. aegypti* has been extensively investigated under laboratory conditions [82–85]. However, relatively little is known about how microclimate impacts the diel host-seeking behaviour of wild *Aedes*. In general, the host-seeking activity *Ae. aegypti* and *Cx. quinquefasciatus* was higher on days when mean temperatures were lower (across the range of 25–30 °C). Additionally, the hourly biting rates of *Aedes* were negatively associated with temperature but only under conditions of low humidity. As mean hourly temperatures were strongly negatively correlated with relative humidity (Additional file 2: Figure S1), these results indicate that *Ae. aegypti* biting activity is highest during relatively cool and humid hours of the day. These microclimatic associations may account for the observed biting activity of *Ae. aegypti* and *Cx. quinquefasciatus.* A comprehensive review [69] of *Ae. aegypti* biting behaviour indicates that bimodal and trimodal activity patterns are often reported, with evidence of specific adaptations to other ecological features (e.g. artificial light availability) [69]. Such variability seems to be common and related to optimal humidity and temperature conditions available during such hours [86, 87].

A key feature of any method for estimating EIR is its ability to estimate human-biting rates and infection rates in mosquitoes. While the results here presented indicate that the MET could be used to estimate the human-biting rates, the infection rates could not be measured as none of the *Aedes* mosquitoes collected with either trapping method were positive for arboviruses. Reported rates of arboviruses in *Aedes* vectors are generally very low (0.1–10%) even in high transmission areas (e.g. [88–95]). Thus, failure to detect arboviruses within the relatively small sample size of vectors tested here (e.g. 207 individuals tested in 122 pools) is not unexpected.

Although promising, the MET has a number of limitations relative to the BGS for sampling host-seeking *Aedes*. First, although both trapping methods require a power supply, the current version of the MET requires two 12 V batteries compared to the one required by the BGS), requires human participants and the trap itself is heavier, which is more labour-intensive than using BGS. Also, as the METs used here are still research prototypes produced on a bespoke basis without a licensed manufacturer, their production cost is currently more expensive than BGS traps (approximately £650 *vs* £170 per trap, respectively). In addition, some technical problems were experienced including a tendency to short circuit under conditions of high air humidity. These limitations are expected to be improved if manufactured at scale as manufacturing costs would fall and technical improvements should make the MET suitable for humid environments. The primary advantage of the MET is, therefore, its potential ability to directly estimate the EIR for arboviral infections. This advantage could be leveraged to calibrate other existing trapping methods that are less labour intensive and more feasible to be deployed at large scale. Additionally, the MET could be used in combination with other trapping methods to identify hotspots of transmission before large scale deployment with other traps is carried out.

## Conclusions

Here, we evaluated the MET as a tool for estimating human biting rates of the arboviral vector *Ae. aegypti* in a high transmission setting in coastal Ecuador. The MET performed at least as well as the current BG-Sentinel trap gold standard for estimating the mean abundance per hour of host-seeking *Aedes* and provided a realistic representation of hourly activity patterns. We conclude that MET is a promising tool for *Ae. aegypti* and other mosquito species surveillance, which could uniquely enable a relatively direct estimate of the arboviral entomological inoculation rate experienced by communities.

## Supplementary information

**Additional file 1: Text 1:** Additional Methods. **Table S1.** Primers used for detection of arboviruses by RT-PCR. **Table S2.** Positive control DNA sequences used as PCR positive controls. **Text 2.** Additional Results.

**Additional file 2: Figure S1.** Relationship between observed temperature (°C) and relative humidity (%). Red dots represent individual observations per hour recorded.

**Additional file 3: Figure S2.** Visualization of the PCR products of S7 gene on agarose gels. All samples were positive, except 920-1.

**Additional file 4: Figure S3.** Visualization of the first PCR products of ZIKV on agarose gels. Expected size of positive fragments: 76 bp. ZIKV+: positive control.

**Additional file 5: Figure S4.** Visualization of the first PCR products of DENV 1–3 on agarose gels. Expected size of positive fragments: 63 bp. DENV1+: positive control.

**Additional file 6: Figure S5.** Visualization of the first PCR products of DENV 4 on agarose gels. Expected size of positive fragments: 63 bp. DENV4+: positive control.

**Additional file 7: Figure S6.** Visualization of the second PCR products of ZIKV on agarose gels. Expected size of positive fragments: 76 bp. ZIKV+: positive control.

**Additional file 8: Figure S7.** Visualization of the second PCR products of DENV 4 on agarose gels. All samples were negative. Expected size of positive fragments: 63 bp. DENV4+: positive control.

**Additional file 9: Figure S8.** Visualization of the second PCR products of DENV 1-3 on agarose gels. Expected size of positive fragments: 63 bp. DENV1+: positive control.

**Additional file 10: Figure S9.** Visualization of the individual PCR products of DENV1, DENV2 and DENV3. Samples from Figure S8 are shown for individual runs for each of the three DENV isotypes. Expected size for DENV1 run was 71 bp, 199 bp for the DENV2 run, and 167 bp for the DENV3 run. DENV1+, DENV2+ and DENV3+: positive controls.

## Data Availability

Data supporting the conclusions of this article are included within the article and its additional files. The dataset generated and analysed during this study is publicly available in the Open Science Framework repository at https://osf.io/zwbs8.

## Abbreviations

HLC: human landing catches
EIR: entomological inoculation rate
MET: mosquito electrocuting trap
BGS: BG-sentinel trap
ZIKV: Zika virus
DENV: dengue virus
CHIKV: chikungunya virus
WNV: West Nile virus
GLMM: generalized linear mixed models
LRT: likelihood ratio test
PCR: polymerase chain reaction
