# Supplementary material for: The mosquito electrocuting trap as an exposure-free method for measuring human-biting rates by Aedes mosquito vectors
Source: Parasit Vectors. 2020 Jan 15;13:31. doi: 10.1186/s13071-020-3887-8 (PMC6961254; doi:10.1186/s13071-020-3887-8)
Supplement: Supplementary file 1 — Additional file 1: Text 1: Additional Methods. Table S1. Primers used for detection of arboviruses by RT-PCR. Table S2. Positive control DNA sequences used as PCR positive controls. Text 2. Additional Results. [file 13071_2020_3887_MOESM1_ESM.docx]

**Additional file 1**

**Text S1. Additional Methods**

**RNA extraction, reverse-transcription and PCR**

Mosquito pools were placed in 1.5 ml crio-vials containing TRIzol™ (Invitrogen) and stored at -80°C for RNA preservation at LEMMT-USFQ before being shipped on dry ice to the MRC-University of Glasgow Centre for Virus Research (CVR) for further analysis. At CVR, samples were transferred to a -80 °C freezer until further processing.

To extract RNA, glass beads were added to each mosquito pool and used to homogenize the sample with a Precellys 24™ homogenizer (Bertin Instruments; homogenization for 30 seconds at 6800 rcf in TRIzol™ Reagent). RNA was then extracted from samples using TRIzol™ according to the manufacturer’s instructions; with the exception that 1-Bromo-3-chloropropane (Sigma) being used instead of chloroform. RNA was precipitated with isopropanol and the pellet washed with 75% ethanol and then re-suspended in 50 µl of nuclease-free water. The concentration and quality of the extracted RNA was measured using a NanoDrop 2000™ (Thermo Fisher Scientific). RNA samples were subsequently aliquoted and stored at -80 °C until the reverse-transcription (RT) step.

RTs as well as negative RTs were performed using Moloney Murine Leukemia Virus Reverse Transcriptase™ (M-MLV RT) (Promega) from 950.4 ng of RNA in 40 µl final according to the manufacturer’s instructions. cDNAs were aliquoted and further stored at -20°C until PCR was carried out. Out of 147 pools, the concentration of RNA in 25 was too low for analysis and thus excluded from further processing the analysis.

PCR was performed on cDNA prepared from mosquito pools using the DreamTaq Green Polymerase (ThermoFisher) following manufacturer’s instructions. A first round of PCR was run on all samples to amplify the S7 gene (ribosomal protein) to assess the RNA integrity of the sample (*i.e.* RNA successfully reverse-transcribed into cDNA). For all samples where RT was confirmed to have succeeded, subsequent PCRs were performed to amplify specific cDNA regions to detect Zika (ZIKV), dengue (DENV) (1-4 strains) and chikungunya virus (CHIKV). Primers, melting temperatures and expected sizes used are listed in Table S1. Some published primers were shortened to have the same melting temperature for both forward and reverse primers. To detect the reference gene S7 (ribosomal protein, 35 PCR cycles) and CHIKV, the PCR were run on 1.5% of agarose gel with the DNA ladder used was Gene Ruler 100 bp Plus (Thermo Scientific). While PCR for the other arboviruses (ZIKV, DENV1-3, DENV1, DENV2, DENV3, DENV4, 40 PCR cycles), were run on 3% agarose gel, with the DNA ladder used was Ultra Low Range DNA ladder (Invitrogen). All agarose gels contained EtBr and were visualized with a GelDoc imaging system (Biorad).

Primers used for this study were previously designed and used for virus screening [1–4]. In addition, we checked that they matched relevant strains for each arbovirus to minimize the possibility of missing an infected sample. PCRs for ZIKV, DENV and CHIKV were performed on all of the samples that were sucessfully transcribed into cDNA.

Positive controls were used in all the PCRs performed, therefore cDNAs from experimentally ZIKV-infected *Ae. aegypti* females (infectious blood meal at 10^7^ PFU/mL, whole females 14 days post infection, ZIKV clone pCCI-SP6-ZIKV-Rz [5]) were used as a positive control for ZIKV. For DENV1 and DENV1-3, cDNA from a confirmed infected mosquito with DENV1 was used as a positive control (different field collection, unpublished data). For the rest of the viruses, regions corresponding to the amplified sequence were designed based on previously published virus isolates from GenBank and synthetized (GENEWIZ), diluted at 3 ng/µL and used as positive controls (Table S2).

**Table S1. Primers used for detection of arboviruses by RT-PCR.** Assays included S7 (ribosomal protein) for RNA integrity, Zika virus (ZIKV), universal assay for dengue virus strains 1, 2 and 3 (DENV 1-3), dengue virus strain 1 (DENV1), dengue virus strain 2 (DENV2), dengue virus strain 3 (DENV3), dengue virus strain 4 (DENV4), and chikungunya virus (CHIKV).

| **Assay** | **Primer name** | **Sequence (5’🡪3’)** | **Reference** | **Expected size (bp)** | **Melting temperature** |
| --- | --- | --- | --- | --- | --- |
| S7 | S7 (forward) | GGGACAAATCGGCCAGGCTATC | Newly designed | 403 with intron  290 without | 58°C |
|  | S7 (reverse) | TCGTGGACGCTTCTGCTTGTTG |  |  |  |
| ZIKV | Zika 1087 (forward) | CCGCTGCCCAACACAAG | Modified from [1] | 76 | 57°C |
|  | Zika 1163c short (reverse) | CCACTAACGTTCTTTTGCAG |  |  |  |
| DENV 1-3 | DENV_F  (forward) | GCATATTGACGCTGGGARAGAC | [2] | 63 | 67°C |
|  | DENV_R1-3 | TTCTGTGCCTGGAATGATGCTG |  |  |  |
| DENV1 | DENV1_F  (forward) | CAATGGATGACAACAGAAGAYATG | [3] | 71 | 60°C |
|  | DENV1_R  (reverse) | TCCATCCATGGGTTTTCCTCTAT |  |  |  |
| DENV2 | DENV2_F  (forward) | GCAGAAACACAACATGGAACRATAGT |  | 199 | 60°C |
|  | DENV_2R  (reverse) | TGATGTAGCTGTCTCCRAATGG |  |  |  |
| DENV3 | DENV3_F  (forward) | ATGGAATGTGTGGGAGGTGG |  | 167 | 60°C |
|  | DENV3_R  (reverse) | GGCTTTCTATCCARTAGCCCATG |  |  |  |
| DENV4 | DENV_F  (forward) | GCATATTGACGCTGGGARAGAC | [2] | 63 | 60°C |
|  | DENV_R4  (reverse) | YTCTGTGCCTGGATWGATGTTG |  |  |  |
| CHIKV | CHIKF short (forward) | ACCGGCGTCTACCCATT | Modified from [4] | 312 | 56°C |
|  | CHIKR short (reverse) | GGGCGGGTAGTCCATGTT |  |  |  |

**Table S2. Positive control DNA sequences used as PCR positive controls.** Sequences synthetized with primer sequences in bold. Sequences were designed according to previously GenBank published virus isolates.

| **Assay** | **Positive control Sequence (5’🡪3’)** | **GenBank sequence used** |
| --- | --- | --- |
| DENV1 | GCCCACCAC**CAATGGATGACAACAGAAGACATG**TTATCAGTGTGGAATAG  GGTCTGG**ATAGAGGAAAACCCATGGATGGA**GGACAAAACT | GenBank:  KY474307.1 |
| DENV2 | AAGGAAATA**GCAGAAACACAACATGGAACAATAGT**TATCAGAGTACAATA  TGAAGGGGACGGTTCTCCATGTAAGATCCCTTTTGAGATAATGGATTTGGA  AAAAAGACATGTTTTAGGTCGCCTGATTACAGTCAACCCAATCGTAACAGA  AAAAGATAGCCCAGTCAACATAGAAGCAGAACCT**CCATTCGGAGACAGCT**  **ACATCA**TCATAGGAG | GenBank: AF038403.1 |
| DENV3 | CTCAAGAGC**ATGGAATGTGTGGGAGGTGG**AAGATTACGGGTTCGGAGTTT  TCACAACCAACATATGGCTGAAACTCCGAGAGGTGTACACCCAACTATGT  GACCATAGGCTAATGTCGGCAGCCGTCAAGGATGAGAGGGCCGTACACGC  CGA**CATGGGCTATTGGATAGAAAGCC**AAAAGAATG | GenBank: KU050695.1 |
| DENV4 | ACAAAAACA**GCATATTGACGCTGGGAAAGAC**CAGAGATCCTGCTGTCTCTG  **CAACATCAATCCAGGCACAGAG**CGCCGCGA | GenBank: AY947539.1 |
| CHIKV | AAGGTCTTC**ACCGGCGTCTACCCATT**CATGTGGGGCGGCGCCTACTGCTTC  TGCGACACCGAAAATACGCAATTGAGCGAAGCACATGTGGAGAAGTCCGA  ATCATGCAAAACAGAATTTGCATCAGCATACAGGGCTCATACCGCATCCGC  ATCAGCTAAGCTCCGCGTCCTTTACCAAGGAAATAATATCACTGTGGCTGC  TTATGCAAACGGCGACCATGCCGTCACAGTTAAGGACGCTAAATTCATAGT  GGGGCCAATGTCTTCAGCCTGGACACCTTTCGACAATAAAATCGTGGTGTA  CAAAGGCGATGTCTAC**AACATGGACTACCCGCCC**TTCGGCGCA | GenBank: KR559470.1 |

**Text S2. Additional Results**

All positive controls worked in all of the PCRs carried out in this study. PCRs on S7 showed that RNA was successfully transcribed into cDNA in 121 mosquito pools out of 122 (Figure S2). Weak bands coming from a few samples were observed on the gels from ZIKV, DENV 1-3 (PCR from universal primer) and DENV 4 (Figure S3, Figure S4 and Figure S5). Then, a second PCR was run on only those samples for reconfirmation. Weak bands were observed for a second time in some of the samples of ZIKV (Figure S6a). However, a PCR on the negative RT of those samples also showed weak bands (S6b) suggesting that they may be due to contamination with gDNA. Positivity of DENV 4 could not be confirmed either as no bands were observed on any of the samples (Figure S7). The second PCR of DENV 1-3 showed weak bands for the second time on a few samples (Figure S8), which were then tested on three separated PCRs using individual primers of DENV1, DENV2 and DENV3. However, positivity could not be confirmed either on any of the samples as no bands were observed (Figure S9). PCR results confirmed that no samples were infected with CHIKV either (Figure S10).

**REFERENCES:**

1. Lanciotti RS, Kosoy OL, Laven JJ, Velez JO, Lambert AJ, Johnson AJ, et al. Genetic and serologic properties of Zika virus associated with an epidemic, Yap State, Micronesia, 2007. Emerg. Infect. Dis. 2008;14:1232–9.

2. Alm E, Lesko B, Lindegren G, Ahlm C, Söderholm S, Falk KI, et al. Universal Single-Probe RT-PCR Assay for Diagnosis of Dengue Virus Infections. PLoS Negl. Trop. Dis. 2014;8:6–15.

3. Alm E, Lindegren G, Falk KI, Lagerqvist N. One-step real-time RT-PCR assays for serotyping dengue virus in clinical samples. BMC Infect. Dis. [Internet]. BMC Infectious Diseases; 2015;15:1–7. Available from: http://dx.doi.org/10.1186/s12879-015-1226-z

4. Dayakar S, Goud IK, Remadevi V, Dharmaseelan S, Dayakar S, Nair RR, et al. Molecular Diagnosis of Chikungunya virus (CHIKV) and Dengue virus (DENV) and its concomitant circulation in South Indian population. Virol. Reports [Internet]. Elsevier B.V.; 2015;5:56–62. Available from: http://dx.doi.org/10.1016/j.virep.2015.05.001

5. Mutso M, Saul S, Rausalu K, Susova O, Žusinaite E, Mahalingam S, et al. Reverse genetic system, genetically stable reporter viruses and packaged subgenomic replicon based on a Brazilian zika virus isolate. J. Gen. Virol. 2017;98:2712–24.
