# Supplementary material for: The mosquito electrocuting trap as an exposure-free method for measuring human-biting rates by Aedes mosquito vectors
Source: Parasit Vectors. 2020 Jan 15;13:31. doi: 10.1186/s13071-020-3887-8 (PMC6961254; doi:10.1186/s13071-020-3887-8)
Supplement: Supplementary file 2 — Additional file 2: Figure S1. Relationship between observed temperature (°C) and relative humidity (%). Red dots represent individual observations per hour recorded. [file 13071_2020_3887_MOESM2_ESM.pdf]

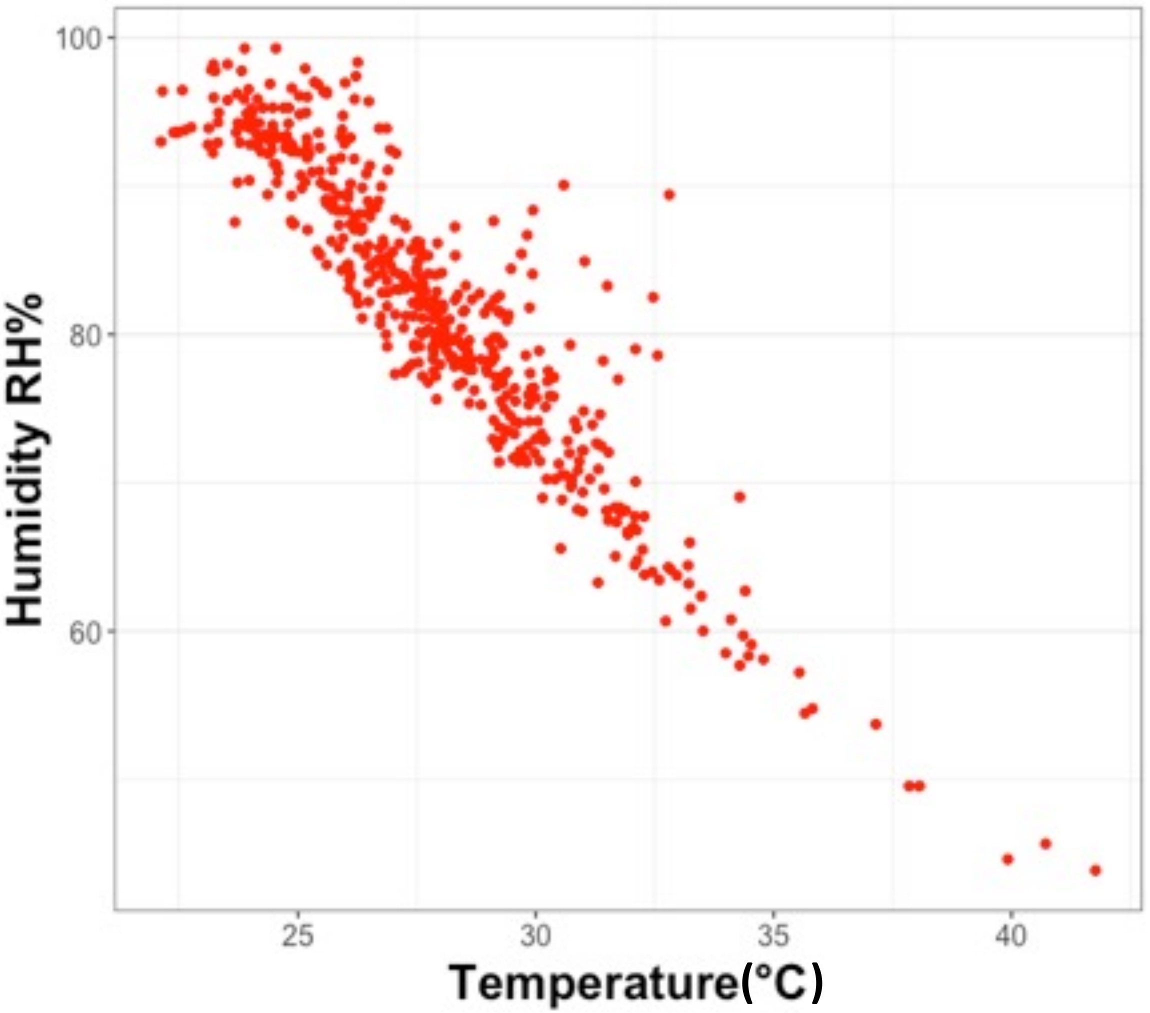

Figure S1. Relationship between observed temperature (°C) and relative humidity (%). Red dots represent individual observations per hour recorded.
