# Supplementary material for: The mosquito electrocuting trap as an exposure-free method for measuring human-biting rates by Aedes mosquito vectors
Source: Parasit Vectors. 2020 Jan 15;13:31. doi: 10.1186/s13071-020-3887-8 (PMC6961254; doi:10.1186/s13071-020-3887-8)
Supplement: Supplementary file 4 — Additional file 4: Figure S3. Visualization of the first PCR products of ZIKV on agarose gels. Expected size of positive fragments: 76 bp. ZIKV+: positive control. [file 13071_2020_3887_MOESM4_ESM.pdf]

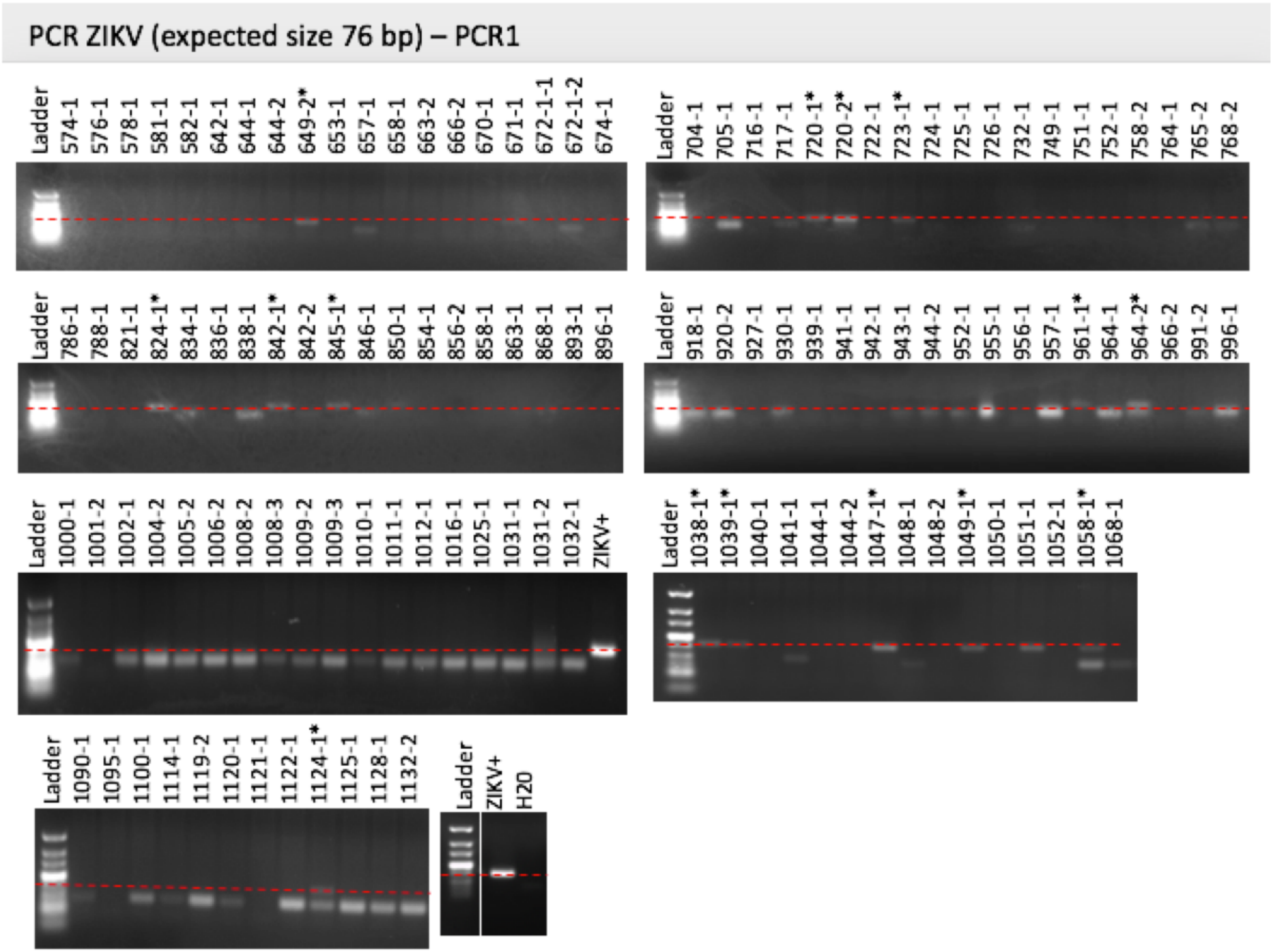

Figure S3. Visualization of the first PCR products of ZIKV on agarose gels. Expected size of positive fragments: 76 bp. ZIKV+: positive control.
