# Supplementary material for: The mosquito electrocuting trap as an exposure-free method for measuring human-biting rates by Aedes mosquito vectors
Source: Parasit Vectors. 2020 Jan 15;13:31. doi: 10.1186/s13071-020-3887-8 (PMC6961254; doi:10.1186/s13071-020-3887-8)
Supplement: Supplementary file 7 — Additional file 7: Figure S6. Visualization of the second PCR products of ZIKV on agarose gels. Expected size of positive fragments: 76 bp. ZIKV+: positive control. [file 13071_2020_3887_MOESM7_ESM.pdf]

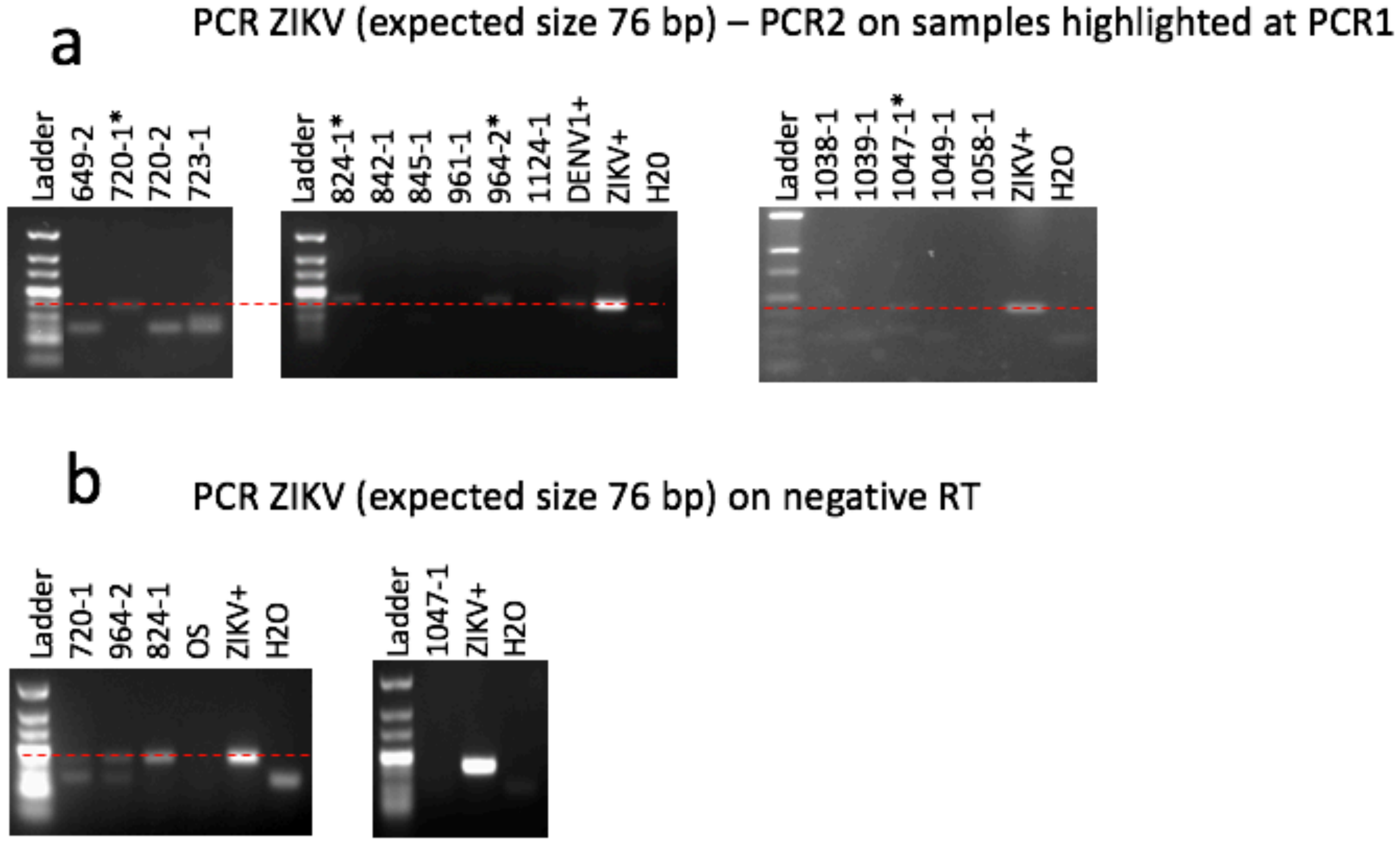

Figure S6. Visualization of the second PCR products of ZIKV on agarose gels. Expected size of positive fragments: 76 bp. ZIKV+: positive control.
