# Supplementary material for: The mosquito electrocuting trap as an exposure-free method for measuring human-biting rates by Aedes mosquito vectors
Source: Parasit Vectors. 2020 Jan 15;13:31. doi: 10.1186/s13071-020-3887-8 (PMC6961254; doi:10.1186/s13071-020-3887-8)
Supplement: Supplementary file 8 — Additional file 8: Figure S7. Visualization of the second PCR products of DENV 4 on agarose gels. All samples were negative. Expected size of positive fragments: 63 bp. DENV4+: positive control. [file 13071_2020_3887_MOESM8_ESM.pdf]

PCR DENV4 (expected size 63 bp) – PCR2 on samples highlighted at PCR1

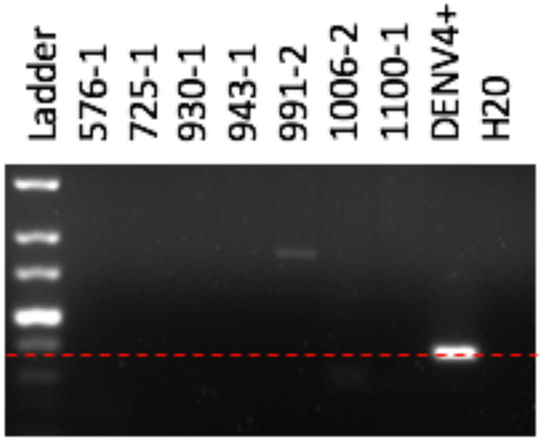

Figure S7. Visualization of the second PCR products of DENV 4 on agarose gels. All samples were negative. Expected size of positive fragments: 63 bp. DENV4+: positive control.
