# Supplementary material for: The mosquito electrocuting trap as an exposure-free method for measuring human-biting rates by Aedes mosquito vectors
Source: Parasit Vectors. 2020 Jan 15;13:31. doi: 10.1186/s13071-020-3887-8 (PMC6961254; doi:10.1186/s13071-020-3887-8)
Supplement: Supplementary file 10 — Additional file 10: Figure S9. Visualization of the individual PCR products of DENV1, DENV2 and DENV3. Samples from Figure S8 are shown for individual runs for each of the three DENV isotypes. Expected size for DENV1 run was 71 bp, 199 bp for the DENV2 run, and 167 bp for the DENV3 run. DENV1+, DENV2+ and DENV3+: positive controls. [file 13071_2020_3887_MOESM10_ESM.pdf]

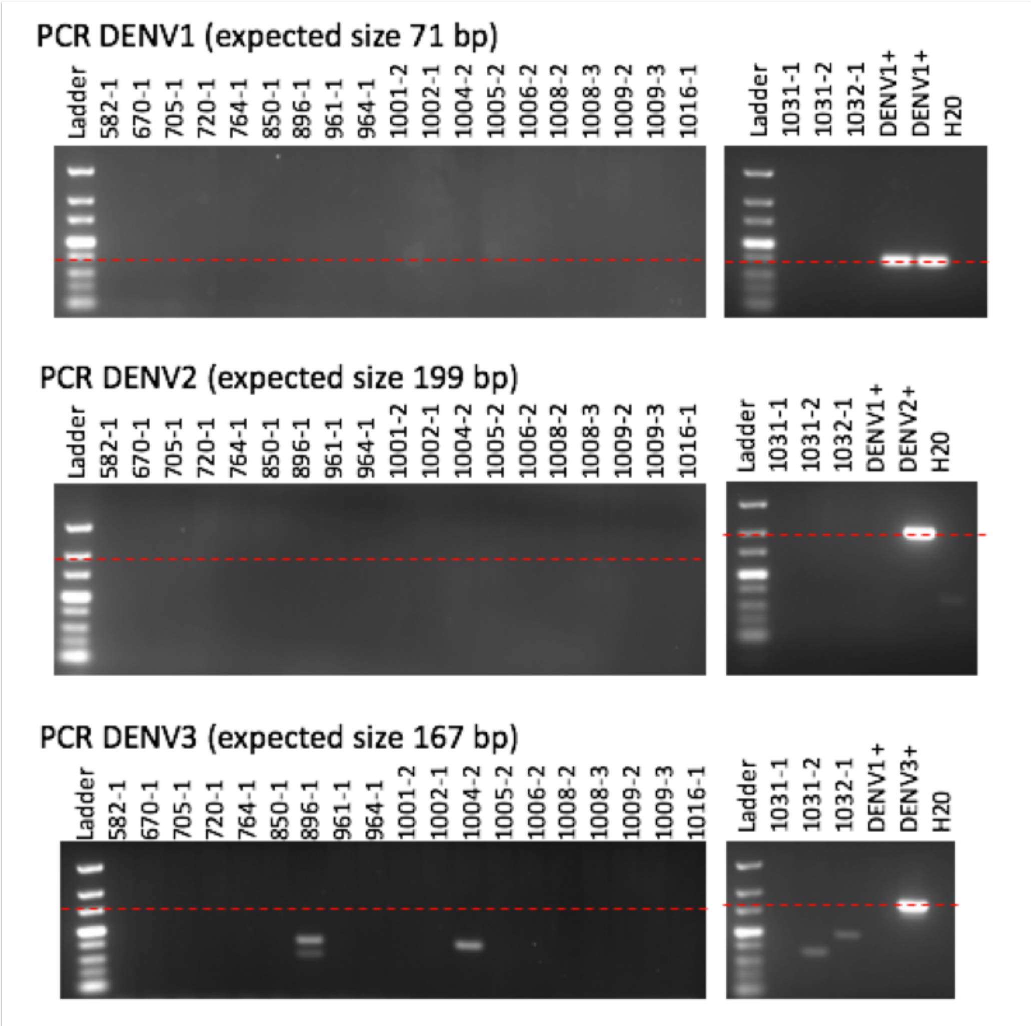

Figure S9. Visualization of the individual PCR products of DENV1, DENV2 and DENV3. Samples from Figure S8 are shown for individual runs for each of the three DENV isotypes. Expected size for DENV1 run was 71 bp, 199 bp for the DENV2 run, and 167 bp for the DENV3 run. DENV1+, DENV2+ and DENV3+: positive controls.
